# Presence of Segmented Filamentous Bacteria in Human Children and Its Potential Role in the Modulation of Human Gut Immunity

**DOI:** 10.3389/fmicb.2018.01403

**Published:** 2018-06-29

**Authors:** Bo Chen, Huahai Chen, Xiaoli Shu, Yeshi Yin, Jia Li, Junjie Qin, Lijun Chen, Kerong Peng, Fei Xu, Weizhong Gu, Hong Zhao, Liqin Jiang, Lanjuan Li, Jian Song, Yoram Elitsur, Hongwei D. Yu, Mizu Jiang, Xin Wang, Charlie Xiang

**Affiliations:** ^1^State Key Laboratory for Diagnosis and Treatment of Infectious Diseases–Collaborative Innovation Center for Diagnosis and Treatment of Infectious Diseases, The First Affiliated Hospital, Zhejiang University School of Medicine, Hangzhou, China; ^2^Department of Gastroenterology, Children’s Hospital, Zhejiang University School of Medicine, Hangzhou, China; ^3^State Key Laboratory of Breeding Base for Zhejiang Sustainable Pest–Key Laboratory for Food Microbial Technology of Zhejiang Province, Zhejiang Academy of Agricultural Sciences, Hangzhou, China; ^4^Promegene Co. Ltd., Shenzhen, China; ^5^Advanced Information Systems, Exelis, Herndon, VA, United States; ^6^Department of Pediatrics, Joan C. Edwards School of Medicine, Marshall University, Huntington, WV, United States; ^7^Department of Biomedical Sciences, Joan C. Edwards School of Medicine, Marshall University, Huntington, WV, United States

**Keywords:** SFB, immunity, SIgA, Th17 cells, ileum microbiota

## Abstract

Segmented filamentous bacteria (SFB) are commensal organisms that grow by anchoring a specialized holdfast structure to the intestinal walls of a variety of animals. Interaction of SFB with Peyer’s patches in mice promotes the post-natal maturation of the immune system. We previously reported that the colonization of SFB in humans mainly occurs by 36 months of age, and is difficult to be detected afterward. In this study, we measured the level of SFB in intestinal fluids of human children. SFB were found via qPCR to represent a small fraction of the whole SFB-positive microbiota (10^5^ SFB in 10^11^ total bacteria). Bacteria with filamentous segmented morphology were observed in intestinal fluids via fluorescent *in situ* hybridization, and from gut biopsies via scanning electron microscopy. SFB-specific DNA and peptide fragments were also identified via multiple displacement amplification PCR and mass spectrometry. There was an overall positive correlation between the presence of SFB and the titer of total secretory immunoglobulin A (sIgA), which is more apparent in intestinal fluids of the age group of 8–36 months. Afterward there was a decline of SFB in numbers correlated with a reduction of total sIgA. RT-qPCR analysis of the terminal ileal biopsies revealed that the expression of Th17 pathway genes were induced in SFB-positive samples, while the markers of T and B cell receptor signaling pathways were also upregulated. Collectively, these data suggest that SFB is a rare member of microbiota, and may play an important role in the development of human gut immunity.

## Introduction

Recently, segmented filamentous bacteria (SFB), or Candidatus *Savagella* (Thompson et al., 2012), have been implicated in the modulation of the host immune system (Ivanov and Littman, 2010). Due to the intimate association with the intestinal epithelium, SFB show the rare ability to induce post-natal maturation of virtually all immune system components. In particular, SFB stimulate differentiation of Th17 cell lineage and T-cell responses in mice (Ivanov et al., 2009). Also, enhanced production of secretory immunoglobulin A (sIgA) was present in SFB-colonized mice (Schnupf et al., 2013). SFB’s ability to induce gut maturation suggests a role in autoimmune diseases such as encephalitis and arthritis. Recent work indicated that co-culture with host cells is essential for cultivation of mouse SFB *in vitro* (Talham et al., 1999), but elucidation of SFB function still relies on this mono-associated mouse model.

Segmented filamentous bacteria have been documented to colonize the guts of various vertebrate animals, including fish, pigs, chicken, mice, and rats (Klaasen et al., 1992), and also display host specificity (Tannock et al., 1984). We previously reported that SFB exist in human feces in an age-dependent manner (Yin et al., 2013). Jonsson (2013) using PCR, also detected SFB in human ileostomy samples and Caselli’s group observed morphological SFB-like Gram-positive bacteria in histological slides of ileo-cecal valves from ulcerative colitis patients (Caselli et al., 2013). Given these pieces of evidence, humans may harbor SFB; however, direct evidence of human SFB is lacking and no other SFB genes, apart from the 16S rRNA gene, have been reported in human specimens. Furthermore, bioinformatics searches of human SFB genes in the Human Microbiome Project (HMP) database and other human metagenomic databases have been negative.

Here, we report that human SFB are present in both the Chinese and US populations. The qPCR and metagenomics analyses of the luminal fluids of children revealed the levels of SFB were extremely low in gut microbiota. Similar to the effect from mouse SFB studies, an enhanced human immune response was observed in the SFB-positive individuals by comparing total sIgA production in the terminal ilea. In addition to the Th17 pathway genes, we found SFB colonization of human terminal ileum is associated with the activation of T and B cell receptor (BCR) signaling pathways.

## Results

### The Concentrations of SFB Are Within a Narrow Range, and Higher in Children Between 21 and 36 Months of Age Relative to Other Age Groups

To investigate the SFB prevalence in American children, we collected a total of 54 fecal samples from 5 day to 15-year-old children. DNA was extracted from the fecal materials and the presence and distribution of SFB was detected via PCR as previously reported (Yin et al., 2013). Children younger than 3 years old showed 68.29% positive identification of SFB in a total of 41 samples (**Table 1**). When age increased to 6 years old, the percentage of positive samples decreased to 50%. However, SFB could be detected in children up to 15 years of age. Additionally, from December 2012 to November 2014, 144 individuals in Hangzhou, China (80 males, 64 females; 2 months to 178 months-of-age) were enrolled in our SFB study (Supplementary Table S1). Luminal fluids from the junction between the cecum and ascending colon were collected via a standard endoscopic procedure. Biopsy specimens from the terminal ilea were also obtained in 54 patients with no apparent clinical symptoms for histopathological examination, scanning electron microscopy (SEM), and/or gene expression profiling (Supplementary Table S1). All samples were first screened for SFB. Sample selection was random within group, and all the human subjects were outpatients free from the inflammation such as IBD or Crohn’s disease but with abdominal pain when admitted.

**Table 1:** Distribution of SFB in fecal samples of US children.

Age (months)	Sample (*n*)	SFB positive (*n*)	SFB^∗^ (%)
0–36	41	28	68.29
37–72	4	2	50
73–108	2	0	0
109–181	6	2	33.33
Total	54	34	64


*^∗^Detected by PCR with SFB specific primers 779F/1008R.*

**FIGURE 1:**
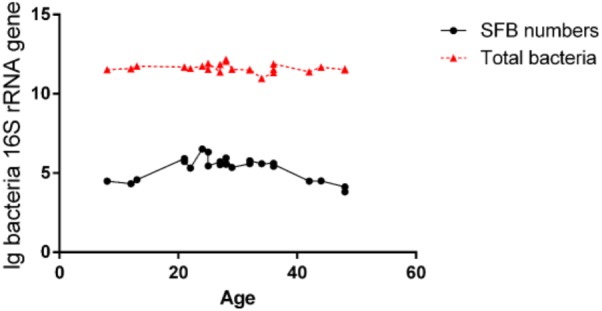
Quantification of SFB abundance in luminal fluids from the terminal ilea of the age group of 8- to 48-month-old of Chinese children. The luminal fluids of disease-free human subjects (n–24) as shown in Supplementary Table S1 (qPCR column) obtained via standard endoscopic procedure were used for the extraction of microbiota DNA. 100 ng amount of DNA per sample was used for qPCR amplification using SFB-specific primers and universal bacterial primers for total bacteria as described in the Section “Materials and Methods.” Comparison of the level of SFB vs. total bacteria.

The luminal fluids were used for the extraction of the microbiota DNA. These DNA samples were tested for the quantification of total bacteria and SFB via qPCR (Supplementary Table S3). We estimated the detection limit of the PCR and qPCR methods was 10^4^ CFU/ml of SFB in an average microbiota of 10^11^ CFU/ml of total bacteria (**Figure 1**). Therefore, the samples below this threshold were considered as SFB-negative. In all SFB-positive samples, the level of SFB was quantified to be between 10^4^ and 10^6^ CFU/ml, but a higher range of concentrations (10^5^–10^6^ CFU/ml) were found in the age group between 21 and 36 months (**Figure 1**). As the ages increased to 36 months, the concentration dropped to 10^4^–10^5^ CFU/ml or below the threshold for detection.

### Identification of Bacterial Structure Similar to SFB With Attachment to the Human Intestinal Walls

**FIGURE 2:**
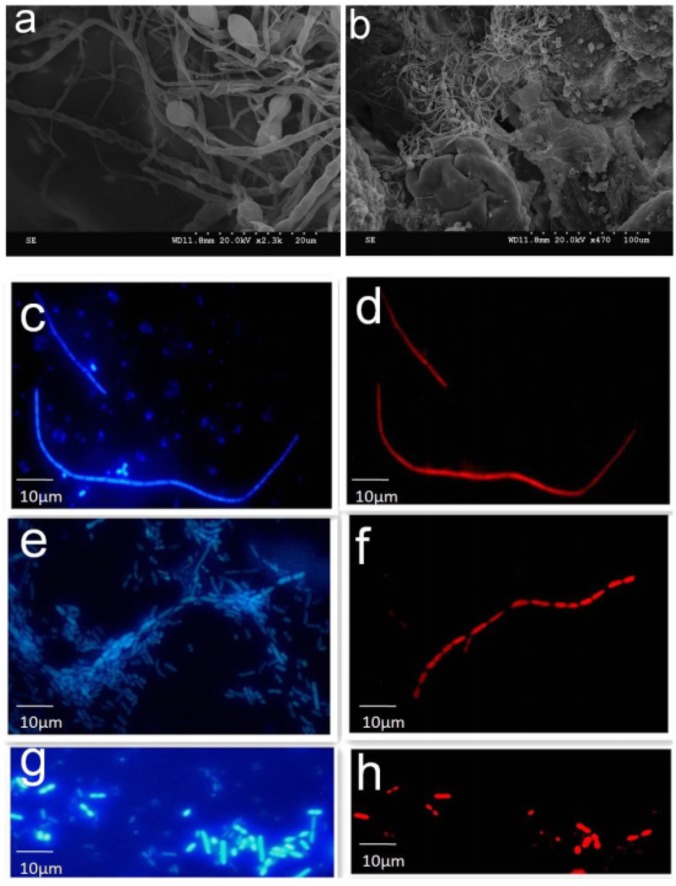
SEM and FISH micrographs of human ileal biopsies. The specimens were processed for the observation of microbiota by SEM and FISH as described in the Section “Materials and Methods.” Samples processed for SEM were labeled in the SEM column in Supplementary Table S1. Terminal ilea of patient 4 **(a,b)** observed under SEM with magnification of 2,300 and 470, respectively. SFB-like structures as visualized with DAPI staining **(c,e)** and hybridization with SFB-specific probe **(d,f)** of patient 4 and 20 (long filamentous shape) observed under epi-fluorescent microscope with magnification of 1,000; SFB by DAPI staining **(g)** and hybridization with SFB-specific probe **(h)** of patient 22 (short rod shape).

To examine the possible interaction of SFB-like structures with intestine, we used two methods, SEM of tissue biopsies and FISH of intestinal fluids using SFB-specific probe. SEM of samples collected from the terminal ilea showed a filamentous shape with variable lengths with a distinct structure that formed septa between two daughter cells (**Figures 2a,b**). However, we did not observe typical holdfast segments. Previously, SFB were identified by their unusual morphology in the terminal ilea of mouse, rats, chicken, fish, and pigs with filamentous shapes (Klaasen et al., 1993a). Our SEM results of SFB morphology are in agreement with previous observations.

Segmented filamentous bacteria morphology in the luminal fluids was also analyzed via FISH (Supplementary Table S2). The SFB probe specificity was previously confirmed (Yin et al., 2013). Bacteria that hybridized positively with the SFB probe had varied morphology; for example, both long typical filamentous shape with segments (**Figures 2c–f**) and short rods (**Figures 2g,h**) were observed in the gut luminal fluids of SFB-positive individuals. Both structures were found in healthy individuals as indicated in Supplementary Table S1.

### Identification of Human SFB-Specific DNA and Protein Fragments in Human Intestinal Fluids

Despite the availability of mouse and rat SFB genomes, the only sequence of human SFB in NCBI database is a partial sequence of the 16S rRNA gene. In order to detect the human SFB-specific DNA, we developed a simple bioinformatics approach. Briefly, all SFB conserved genes were identified by sequence homology of SFB-rat-Yit genes to all other 10 SFB genomes in database. Genes that had some homology to any available genomes of the *Clostridium* spp. were subtracted. Among the 441 SFB conserved genes, 19 top ranking genes were selected for PCR analysis based on their conservation and primers were subsequently designed using Primer3 software (**Table 2**). The DNA was subjected to the enrichment via multiple displacement amplification (MDA). Using this enriched DNA as template, we performed PCR with the primers as listed in Supplementary Table S2. As shown in **Table 2**, PCR products generated by SFB33013 and R49716 belonged to two non-16S genes of Candidatus *Savagella* by comparison to rat and mouse SFB genomes, which encode the homologs of the penicillin-binding protein 2 and the flagellar basal-body rod protein FlgG, respectively. We next used two SFB-positive ileal fluids to test whether the SFB-specific protein fragments can be detected. As shown in **Table 3**, a total of 18 SFB-specific protein fragments were identified by mass spectrometry.

**Table 2:** Detection of human SFB-specific DNA in the gut luminal fluids via PCR.

Primers	Human luminal fluids		
	
	Blast results in NCBI	SFB-positive^∗^ (*N* = 30)	SFB-negative^∗^ (*N* = 28)
SFB33013	*Candidatus Arthromitus* sp. SFB-mouse-Yit (99.4%)^∗∗^	29	1
SFB12314	Cloning vector p7Z6	3	3
SFB8576	No similarity found	30	3
SFB10797	Uncultured organism clone 1041059767815	ND	ND
SFB10788	*Bacteroides fragilis* YMC00/6/496	ND	ND
SFB130710	*Escherichia coli* UMNK88	6	5
SFB81511	*Parabacteroides distasonis* ATCC 8503	ND	ND
R94716	*Candidatus Arthromitus* sp. SFB-mouse-Yit^∗∗∗^	12	0
R49017	No similarity found	2	0


*^∗^Measured by PCR with SFB 16S-specific primers. ^∗∗^SFB33013 PCR product has 949 bps with 943/949 identities to MOUSESFB_0388 encoding the penicillin-binding protein 2 (GenBank: AP012209.1). ^∗∗∗^R94716 PCR product has 762 bps with 756/762 identities to MOUSESFB_1031 encoding the flagellar basal-body rod protein FlgG (GenBank: AP012209.1).*

**Table 3:** Identification of human SFB protein peptides using the gut luminal fluids by mass spectrometry.

No.	Unique peptide	Protein name
1	GVIEEAISEINLELEER	Excinuclease ABC subunit B
2	QKEIFIEFEEDGEYMYFLFHRDK	Hypothetical protein
3	EIFDGEMGIYAIHAGVECGIIK	Aminoacyl-histidine dipeptidase
4	VYSGVTINNVDVSGLSR	Vancomycin B-type resistance protein VanW
5	NFETGTLGDLIK	DUF4214 domain-containing protein
6	EMMDQPEFK	DUF4214 domain-containing protein
7	LNEEIKEIVAR	Type I restriction-modification system subunit M
8	LVVAVSNAHYIEK	PolC-type DNA polymerase III
9	DYKEIEDVIKEIYK	PolC-type DNA polymerase III
10	VADSMFLNFWWTTNR	Endo-beta-*N*-acetylglucosaminidase
11	EKTDGVELQVGANK	Flagellar biosynthesis protein FliC
12	DRNLDVTISIVDR	Flagellar biosynthesis protein FliC
13	AILSEGVELIK	Protein kinase
14	KLEKESNYVLK	DNA ligase (NAD(+)) LigA
15	EKIEVMPVEYEK	Carboxyl-terminal processing protease
16	SLVEVPSDNDLEK	ATP-dependent protease, Lon family
17	RSMVEKNGK	Fe-S cluster assembly protein SufB
18	KSGLDQLIVK	GTP-binding protein YchF


### SFB Is Correlated With Total sIgA Concentration in Luminal Fluids of Human Terminal Ilea

**FIGURE 3:**
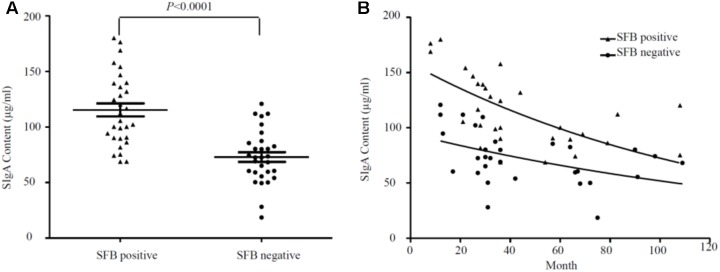
**(A)** Overall relationship between the level of sIgA in luminal fluids from the SFB-positive and SFB-negative patients. **(B)** Relationship between the level of sIgA and the ages of children. A total number of 47 samples (23 SFB-positive and 24 SFB-negative) were used. 500 μl of luminal fluids from each patient were used for the measurement of sIgA as described in the Section “Materials and Methods.” The supernatants were assayed for total secretory IgA using a human IgA ELISA Detection Kit as described in the section “Materials and Methods.” The sIgA was expressed as μg/ml of fecal materials. *P*-values were calculated using Student’s *t*-test.

Segmented filamentous bacteria have recently been identified as an important member of commensal bacteria that can manipulate the host immune system, particularly by inducing strong and broad IgA responses (Schnupf et al., 2015). Mono-association of germ-free mice with SFB colonization increased not only the production of SFB-specific IgA, but also induced non-specific total IgA production equivalent to that of the conventional mice (Klaasen et al., 1993b). Thus, we measured sIgA with ELISA in the luminal fluids (*n* = 47). Samples were divided into SFB-positive and -negative groups based on PCR performed on age-matched specimens (Supplementary Table S1). We observed higher titers of sIgA in SFB-positive samples (118.60 ± 6.65 ± 32.23 μg/ml, median ± SEM *n* = 23, median age: 33.81-month-old) vs. SFB-negative samples (69.21 ± 3.87 ± 23.91 μg/ml median ± SEM, *n* = 24 median age: 34.45-month-old); (*p* < 0.0001) (**Figure 3A**). The relationship between the level of sIgA to the ages of these children were analyzed, and a negative trend was noted in **Figure 3B**. It seems that the majority of elevated levels of sIgA was clustered to the group before the age of 36 months. Surprisingly, we also noticed that IgA decreases with age in the gut independently of whether there is or not SFB, which is particularly obvious after 36 months of age.

### SFB Numbers Are Correlated With the Expression of Th17 Pathway Genes in Human Terminal Ilea

**FIGURE 4:**
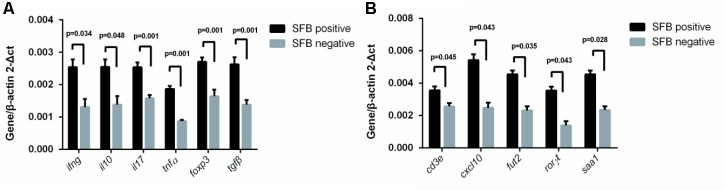
Effect of SFB colonization on Th17 pathway gene expression in human terminal ilea specimens. The tissue biopsies of the terminal ilea from a total of 22 human subjects (11 SFB-positive and SFB-negative, Supplementary Table S1) were used for the extraction of mRNA. 500 ng amount of mRNA was used for RT-qPCR with primers for the Th17 pathway markers as indicated in Supplementary Table S2. **(A,B)** are a select group of Th17 pathway genes used for analysis.

Previously, SFB colonization in the small intestine of mouse can induce the differentiation of naïve T cells into Th17 cells (Gaboriau-Routhiau et al., 2009; Ivanov et al., 2009). The cytokines and transcriptional factor that promote such differentiation include IL-23, IL-17, TGF-B, and ROR-γ (Bettelli et al., 2006; Ivanov et al., 2006; Mangan et al., 2006; Veldhoen et al., 2006). To identify the effects of SFB colonization on the expression of human Th17 pathway genes, we extracted mRNA from a total of 22 human biopsies (SFB positive samples *n* = 11 vs. SFB negative samples *n* = 11) without any clinical inflammation and gross pathology. The qRT-PCR was performed to assay the gene expression of Th17-related pathway markers. The results in **Figure 4** demonstrates that *IFN*-γ, *IL-10*, *IL-17*, *foxp3, tgf*-β, *ror*-γ, and *TNF*-α expression was significantly enhanced in SFB-positive samples relative to SFB-negative samples, indicating the expression of Th17 pathway genes likely were affected in SFB-positive human ileal samples. We also measured expression serum amyloid A (SAA) and galactoside 2-alpha-L-fucosyltransferase 2 (Fut2), because both are epithelial genes and SAA production is regulated by host IL-17 (Anthony et al., 2013; Conti and Gaffen, 2015). **Figure 4B** shows that SAA and Fut2 expression was significantly different between SFB-positive and SFB-negative specimens. Collectively, these data show that SFB colonization in human ileum likely affects differentiation and maturation of multiple CD4+ T helper subsets.

### SFB Colonization in Human Terminal Ilea Is Associated With Activation of T and B Cell Receptor Signaling Pathways

**FIGURE 5:**
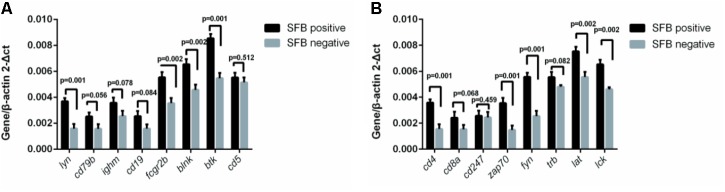
Effect of SFB colonization on the induction of T and B cell receptor signaling pathways in human terminal ilea biopsies. The 22 biopsies were collected and used for the analysis of the gene expression of a select group of receptor pathway genes. Data are expressed as the mean ± SD (*n* = 22). *P*-values were calculated using the Student’s *t*-test. RT-qPCR was performed as described in the Supplementary Materials and Methods. **(A,B)** are a select group of BCR and TCR signaling pathway genes, respectively.

To investigate SFB–host interactions and the host response to SFB, we compared the expression profiles in terminal ileal mucosa from three SFB-positive and three SFB-negative age-matched individuals. All these tissue biopsy specimens were collected from the location where clinical inflammation and gross pathology are absent but have abdominal pain. They micro-array slides used for hybridization contain roughly 42,545 GenBank accessions (GSE68425). When we performed two group comparison (SFB positive samples *n* = 3 vs. SFB negative samples *n* = 3) using the standard pipeline Agilent Genespring GX, only six out of 42,545 genes entitled satisfying corrected *p*-value cut-off 0.05 (Supplementary Table S3). These six samples are from age closely matched, healthy individuals (Supplementary Table S1, the chips column). This suggests that there are significant variations in the gene expression profiles within group. However, when comparing person to person, metallothionein (MT)-Heavy Metal, T cell receptor (TCR) and BCR signaling emerged as significant pathways in most of comparisons (Supplementary Table S4), suggesting the samples of the human terminal ileal biopsies used in the current study are highly heterogeneous. This could be due to the fact the tissues contain a mixed population of cells (T cells, B cells, epithelial cells, etc.) and/or a significant variation in the gene expression profiles of these cell types from person to person. Furthermore, most B cells in the gut are terminally differentiated plasma cells. But since SFB can induce the Th17 differentiation, we further validated the T and BCR signaling pathways. We performed qRT-PCR to assay the levels of eight genes in each signaling pathway in Supplementary Table S5 using the same mRNA samples of **Figure 4**. Seven out of eight TCR pathway markers are significantly elevated (**Figure 5A**). These include CD4, CD8A, ZAP70, FYN, TRB, LAT, and LCK. ZAP70, FYN, TRB, LAT, and LCK encode zeta chain of TCR associated protein kinase 70, FYN proto-oncogene of Src family tyrosine kinase, TCR beta locus, linker for activation of T-cells and LCK proto-oncogene of Src family tyrosine kinase, respectively. CD247 was the only marker whose expression was not verified. Similarly, six out of eight genes were found with significant changes in the BCR pathway, Lyn, CD79B, IGHM, FCGR2B, BLINK, and BTK (**Figure 5B**). These genes encode proto-oncogene of the Src family tyrosine kinase, CD79b molecule, immunoglobulin heavy constant mu, Fc fragment of IgG receptor IIb, B-cell linker, and Bruton tyrosine kinase, respectively. Only two genes CD5 and CD19 that were found not significant in qRT-PCR results (**Figure 5B**).

### SFB Are Associated With Difference in the Gut Microbiota

To examine the effect of SFB on the microbial community of the gut luminal fluids, we carried out next-generation shotgun sequencing of three SFB positive samples and three SFB negative samples from healthy subjects. Each sample was repeated two or three times. Each sample was *de novo* sequenced to produce an average of 4.03 ± 0.88 GB data. A total of 44.28 GB data was pooled for metagenomics analysis. After removing human DNA sequences, we aligned the high quality reads to SFB genomes from NCBI, however, the average genome coverage of SFB was less than 1%, indicating that the sequencing at the level of 4 GB was not sensitive enough to detect the SFB sequence in the microbiota. Thus, we deduced that the abundance of SFB in the microbiota was below 5 × 10^6^, which was consistent with the qPCR results (**Figure 1**).

To understand the difference microbial community structure between SFB positive samples and SFB negative samples, and Shannon index of all individuals from the former seemed higher (Supplementary Figure S1a). Furthermore, a principal coordinate analysis (PCA) showed clustering of samples corresponding to SFB; In the PCA result, several highly contributed genera were *Eubacterium, Prevotella, Roseburia*, and *Bacteroides* (Supplementary Figure S1b).

To assess how SFB affects the microbiota, we compared the composition of microbiota in luminal fluids between SFB positive and negative groups (Supplementary Figure S1c). Linear discriminant analysis (LDA) indicated that the genus *Clostridia, Coriobacteriia*, and *Deltaproteobacteria* were increased in SFB positive samples, while genus *Bacteroides, Escherichia*, and *Klebsiella* were increased in SFB negative samples.

## Discussion

The presence of SFB in the human gastrointestinal tract is debatable, although Jonsson (2013) and Yin et al. (2013) showed SFB can be detected in the intestinal microflora. However, several genome-wide attempts to identify the SFB sequences have failed so far despite a large number of human intestinal metagenomes being interrogated (Prakash et al., 2011; Sczesnak et al., 2011). One of the main reasons is due to the very low amount of SFB in the microbiota. Lagier et al. (2015) reported that in a complex ecosystem that consists of 10^12^ bacteria, current metagenomic studies are unable to detect bacteria at the level of 10^5^ bacteria. Indeed, we didn’t identify SFB-like sequences from 44.28G metagenomics sequences of the microbiota. The amount of SFB, as an immune-modulator of the small intestine in mice, is tightly regulated. There is a balanced SFB-containing microbiota in the gut of normal mice. The overgrowth of SFB in mice has been linked with autoimmune diseases (Van Praet et al., 2015). Overgrowth has also been shown through the disruption of the immune pathways such as the knockout of IL-17 receptor or the suppression of the IL-17A and neutrophil in the gut (Kumar et al., 2016). These observations are consistent with current observation. Interestingly, in humans, the peak of SFB abundance coincides with a period of 21–36 months of age, which is thought to be important for the post-natal development of the immunity (Flannigan et al., 2016). However, in our study, we didn’t observe the typical holdfast morphology through SEM. Transmission electron microscope (TEM) may provide much better resolution for visualization of this specialized structure connecting to the host epithelium.

The percentage of SFB positivity in American children before 36 months of age was 68.3 % (**Table 1**), which is higher than 46.1% from the same age group of Chinese children (Yin et al., 2013). This may be due to difference in where these samples are collected. The samples of our previous study came from a daycare setting while the samples of the current study are all from outpatients. Additionally, we identified two American children with SFB colonization whose ages are up to 15 years old. But due to the small sample size, this number may not truly reflect the actual percentage of SFB colonization in this age group. We can also not rule it out whether these two patients may have underlying intestinal diseases. However, in our previous survey, we noted the SFB colonization can occur in young adult group (Yin et al., 2013).

We and others previously used 16S-derived primers to detect human SFB. Since bacteria normally have multiple copies of rRNA genes in the genome, the advantage of using 16S primers is they facilitate detection of the low-concentration target such as SFB. However, the problem is the 16S rRNA genes are highly conserved. It is not clear how specific these primers are for SFB. To circumvent these problems, we identified two specific genes for human SFB (**Table 2**). These genes showed almost identical sequences to mouse SFB homologs. Together with the proteomic data from the luminal fluids of human ilea, we conclude that human SFB exist in the intestinal mucosa. To identify these non-16S rRNA genes, we used an MDA procedure to enrich the microbiome for SFB detection, which otherwise is below the detectable level using regular PCR. These additional data support the notion that the level of human SFB is low in the microbiota.

Although human SFB is low in population, its colonization seems to affect the diversity and composition of the microbiota. *Eubacterium, Prevotella*, and *Roseburia* are the most predominant genera of bacteria in the SFB positive intestinal microflora, while *Bacteroides* is the most abundant in the SFB negative microflora. Interestingly potential pathogenic bacteria such as *Escherichia* and *Klebsiella* were increased in the negative group, which is in concordance with the observation of the microflora in mice. Ivanov et al. (2009) reported that the colonization of SFB in mice is able to reduce the infection of the murine colon pathogen, *Citrobacter rodentium*. The fact that SFB colonization competitively exclude pathogens from the distal small intestine have been reported as early as 1982 by Garland et al. (1982). Obviously, the stimulation of sIgA by the colonization of SFB is considered as one of the mechanisms for resistance to pathogen colonization (Jiang et al., 2001).

The total sIgA response at the intestinal mucosa is one of the primary defense mechanisms protecting against enteric infections (Rogier et al., 2014) and modulation of bacterial colonization through dietary supplementation with probiotics or prebiotics could lead to the increase of total fecal sIgA (Campeotto et al., 2011; Roze et al., 2012). In the current study, total fecal sIgA was significantly elevated in the SFB positive group compared to the negative group, suggesting the colonization of SFB may affect the activation of humoral immune responses. Similar observations regarding the induction of sIgA by the colonization of SFB is well documented in mice (Jiang et al., 2001). In an extreme scenario, the transgenic mice lacking the enzyme to make sIgA has a selective overgrowth of SFB in gut microbiota (Jiang et al., 2001). However, it has been reported that sIgA have a stimulatory effect on the presence of SFB (Klaasen et al., 1993b), which supports our observation about the association of an elevated level of sIgA with a high number of SFB during the neonatal development. Pathway associated with B-cell receptor activation were up-regulated in the SFB-positive samples relative to the SFB-negative samples, suggesting that SFB colonization is involved not only in T cell, but also B-cell development.

The induction of T and B cells by SFB colonization is a hallmark for previous SFB studies, particularly for the induction of Th17 cell proliferation in mice (Ivanov et al., 2009) SFB adhesion to the intestinal epithelial cells is considered to be integral for the induction of Th17 activation (Atarashi et al., 2015). In the current study, we observed attachment of SFB-like filamentous bacteria through SEM of biopsies to the surface of distal ileal epithelial cells. However, our FISH results also showed that SFB can exhibit a rod-like structure in the luminal fluids without attachment to the epithelium. This rod shape of SFB has an apparent relationship to pathology. Additionally, we documented markers suggestive of an increased T and B cell response when human ileal samples were colonized with SFB. A range of genes, including *TNF*α, *Il-10*, *CXCL10*, *INF*γ, *CD3E*, *Foxp3*, *GABBR2*, and *FCAMR* shown to be up-regulated in mice by the colonization of SFB, were also up-regulated in the current study with human terminal, SFB-positive ileal biopsies, suggesting that human SFB plays a similar role in the immune stimulation (Ivanov et al., 2009). Meanwhile, it is interesting to note that the expression level of IL-17 production is significantly elevated in SFB-positive ileal biopsies that are in line with the mouse data, implying a similar pattern of immune response development shared by mouse and human in response to SFB colonization.

In conclusion, SFB are present in human intestinal fluids as a low fraction of the whole microbiota. Identification of SFB-specific DNA and peptide fragments in human ileal fluids provides additional evidence that human gut harbors SFB. Previous published data indicate that SFB triggers the post-natal maturation of the intestinal immune system in mice (Ivanov et al., 2009; Schnupf et al., 2013; Atarashi et al., 2015). A similar trend in the total sIgA and Th17 pathway marker expression in SFB-positive individuals suggests that the immune stimulation pathways by the colonization of SFB may be conserved between mice and human. It is also possible that the altered levels of immunological markers as observed in this study are due to other bacteria other than SFB since the composition of two microbiotas with/without SFB is different. Since SFB is only a minority member of the human gut microbiota, its role in the activation of inflammatory pathways in the human gut needs to be further investigated.

## Materials and Methods

### Colonoscopy and Sample Collection

Both studies were approved by the Committee of Ethics at the Children’s Hospital, Zhejiang University School of Medicine, and Marshall University Joan C. Edwards School of Medicine. All patients were informed ahead of time of all possible outcomes and adverse events following such a procedure and their samples would be used in this study in a de-identified manner. After bowel preparation, patients were given 10% chloral hydrate (0.5 ml/kg, po) and sedation with midazolam (0.1–0.2 mg/kg, iv) 30 min before the colonoscopy. Luminal fluids at the cecum and ascending colon were collected and several specimens from terminal ilea were obtained when the colonoscope was extended to the distal ileum for routine histopathological examination. Tissue specimens were immediately frozen in liquid nitrogen and stored at -80°C for further analysis. The sample information was listed in Supplementary Table S1.

### Sample Selection

Endoscopic biopsy samples weighted around 10–15 mg per piece, with 2–3 pieces acquired from each patient, and combined weight of these species generally less than 50 mg per sample. First, we performed PCR screening for SFB in all 144 samples. To confirm the presence of SFB by SEM and FISH, we tested 36 SFB positive samples based on the availability of sample materials.

For CHIPS: Since SFB were detected in 6–36 months more frequently, we choose three SFB positive and three negative samples randomly from these children. For measurement of sIgA: 23 samples chosen by random from 36 SFB positive samples, and 24 samples randomly chosen from SFB negative samples. Again, this was done based on the amount of sample materials available.

mRNA: 36 SFB positive samples, and 11 samples all randomly chosen from SFB negative samples.

QPCR and non-16S gene: 24 randomly chosen from 36 SFB positive samples.

Meta sequence: Seven samples randomly chosen from 36 SFB positive samples, and four randomly chosen from SFB negative samples.

### Scanning Electron Microscopy (SEM)

Scanning electron microscopy was used to view 0.2 mm × 1 mm samples from the terminal ilea. Biopsies were fixed in 4% glutaraldehyde in 0.2 M cacodylate buffer, and processed as described in the literature (Liao et al., 2012). Biopsies of terminal ilea obtained from endoscopy were immediately fixed in 4% glutaraldehyde-cacodylate buffer, dehydrated and processed as described in the literature (Gaboriau-Routhiau et al., 2009; Liao et al., 2012). Samples were observed under an H-9500 Hitachi SEM microscope at 20 kV after coating samples with gold-palladium film.

### FISH Procedure

Luminal fluids were centrifuged at 12,000 × *g* and pellets were fixed in 4% paraformaldehyde solution as previously described (Liao et al., 2012). Samples were stored in 50% ethanol at -20°C and fixed bacteria were spotted onto glass slides and allowed to air-dry for 1 h. An aliquot of 20 μl hybridization solution (0.9 M NaCl, 20 mM Tris/HCl, 0.01% SDS, pH 7.2) containing 5 ng SFB-specific oligonucleotide probe (Supplementary Table S2) was spotted onto the air dried cells and incubated overnight at 50°C (Zarda et al., 1991). After slides were immersed in washing solution (0.9 M NaCl, 20 mM Tris/HCl, 0.01% SDS, pH7.2) at 48°C for 15 min, slides were briefly rinsed with sterile water, air-dried, and then stained with 4′,6-diamidino-2-phenylindole (DAPI) for 15 min. After washing with 80% ethanol and sterilized water, air-dried bacteria cells were observed and photographed using an Olympus epi-fluorescent microscope CX-51 (Olympus Corporation, Japan).

### DNA Extraction and PCR Amplification With SFB Specific Primers

Bacterial genomic DNA from luminal fluid was extracted using the QIAamp DNA Stool Mini Kit according to the manufacturer’s instructions (Qiagen, German). DNA was quantified using a NanoDrop ND-2000 (NanoDrop Technologies, Wilmington, DE, United States), and its integrity and size was confirmed by agar gel electrophoresis (1.0%). Oligonucleotides targeted to the SFB 16S rRNA gene region (779F/1008R, Supplementary Table S2) were used as PCR primers. Standard PCR was performed as described in the literature (Yin et al., 2013). Amplification products were separated using TAE agarose gel electrophoresis (1.0%) and bands were visualized under UV light.

### Quantification of SFB in Luminal Fluids by qPCR

Quantification of bacterial DNA was performed using an ABI PRISM 7500 Real-Time PCR Detection System (Applied Biosystems) according to the manufacturer’s instructions. A 20 μl amplification reaction was performed with 10 μl Thunderbird SYBR quantitative PCR (qPCR) Mix (Toyobo Co., Ltd., Osaka, Japan), 0.04 μl 50 × ROX reference dye, 0.5 mM of each primer, 1 μl DNA template (20 ng/μl), and distilled water. Amplifications were performed as follows: one cycle at 95°C for 1 min, 40 cycles at 95°C for 15 s, at appropriate annealing temperatures for 35 s and 72°C for 35 s. Fluorescence was measured after the extension phase of each cycle. Melt curve analyses were performed by slowly heating the PCR mixtures from 55 to 95°C. These served as end point assays and were used to confirm PCR specificity. Primers for total number of bacteria and SFB were selected for quantification and the sequences are presented in Supplementary Table S2. The quantitative measurement of unknown samples was achieved using standard curves made from known concentrations of plasmid DNA containing the respective amplicons for each set of primers (Fite et al., 2004).

### DNA extraction, Library Construction, and Sequencing

The luminal fluid sample DNA libraries were constructed using Illumina TruSeq DNA Sample Prep Kit according to the manufacturer’s instruction. Illumina TruSeq PE Cluster and SBS Kit were used to perform cluster generation, template hybridization, isothermal amplification, linearization, blocking and denaturization and hybridization of the sequencing primers. Paired-end sequencing 2× 100bp was performed to sequence all libraries. The base-calling pipeline (Casava 1.8.2 with parameters—use-bases-mask y100n, I6n, Y100n, –mismatches 1,–adapter-sequence) was used to process the raw fluorescent images and all sequences (Qin et al., 2015).

### Enzyme-Linked Immunosorbent Assay (ELISA)

To measure total sIgA concentrations in the luminal fluid, approximately 500 μl samples were centrifuged for 20 min at 1,000 × *g* at 4°C. Collect the supernatant and carry out the assay immediately. The 96 plates were washed in PBS and blocked in PBS with 1% BSA. Samples were diluted 1/10, and a twofold serial dilution was made. Samples were incubated at room temperature for 2 h. Total sIgA was quantified by ELISA using a human secretory IgA ELISA detection Kit (Elabscience, Wuhan, China). The measurement was taken according to the manufacturer’s instructions.

### RNA Extraction and Gene Expression Analysis

Biopsy specimens of terminal ilea were lysed in Trizol (Life Technologies, Carlsbad, CA, United States) according to the manufacturer’s instructions and total RNA was extracted and purified by using RNeasy mini Kit (Qiagen, GmBH, Germany). RNA samples of each group were then used to generate fluorescent-labeled cRNA targets for the Agilent Whole Human Genome Oligo Microarray (4 × 44K). Labeled cRNA targets were then hybridized with the slides and slides were scanned on an Agilent Microarray Scanner (Agilent technologies, Santa Clara, CA, United States). Data were extracted with Feature Extraction software 10.7 (Agilent technologies, Santa Clara, CA, United States). Raw data were normalized using a quantile algorithm (GeneSpring Software 11.0, Agilent technologies, Santa Clara, CA, United States) and subsequently adjusted for multiple testing by the Benjamini and Hochberg false discovery method (*p* < 0.05). Microarray experiments were performed using an Agilent Technologies Inc.

### Bioinformatics Analyses for Microarray Assay

Ratios were calculated between SFB-positive and SFB-negative patients. Genes with at least a twofold change were selected for further analysis and selected genes were grouped in functional categories using the standard pipeline Agilent Genespring GX and processes were selected based on *P*-values smaller than 0.05.

### Quantitation of Gene Expression With Real-Time PCR

RNA was purified from intestinal tissues and RT-qPCR was performed with either the TaqMan gene expression assay with TaqMan Universal PCR master mix (Applied Biosystems) or human-specific primers with SYBR-Green PCR master mix (Applied Biosystems). The *cd3e*, *cxcl10*, *IFN*γ, *IL-10*, *IL-17*, *TNF*α, *foxp*3, *Fut2* and SAA primer sequences are listed in Supplementary Table S2. cDNA samples were assayed in duplicate and gene expression for each sample was normalized relative to actin with a 2^-Δ*C*_t_^ calculation.

### Bacterial Protein Digestion and SFB Peptide Identification

Ileal wash fluids from two individuals were used for bacterial protein extraction. After gel electrophoresis, five protein bands with molecular weight ranged from 40 to 50 kDa were excised for LC-MS analysis. In-gel digestion, peptide extraction and desalting were performed as described previously (Shevchenko et al., 1996; Rappsilber et al., 2007). The eluted peptides were dried and dissolved with sample loading buffer (1% ACN and 1% formic acid in water) for MS analysis. The peptide identification was performed on a Q Exactive HF mass spectrometers (Thermo Fisher Scientific, San Jose, CA, United States) equipped with a Ultimate 3000 ultra-performance liquid chromatography (UPLC) system (Thermo Fisher Scientific, San Jose, CA, United States). The raw peptide data were submitted to Sequest HT server using Proteome Discoverer software (Thermo Fisher Scientific, San Jose, CA, United States). Local database were builded by downloaded SFB protein sequences from UniProt^1^. SFB peptides were identified using search parameters consisting of peptide mass tolerance range from -15 to 15 ppm, fragment mass tolerance 20 mmu, peptide confidence high, peptide length ≥4, and peptide FDR ≤ 0.01. Peptides with up to two missed cleavages were allowed. The SFB unique peptides were identified using NCBI BLAST^2^ analysis. The coverage and identity with 100% matching to mouse SFB proteins only were considered as SFB unique peptide.

### Data and Materials Availability

All mRNA sequences used were deposited in the National Center for Biotechnology information Gene Expression Omnibus with accession number GSE68425. The raw Illumina read data of all samples has been deposited in the NCBI Sequence Read Archive under accession numbers SRP069299.

### Statistics

Significance was scored with an unpaired Student’s two-tailed *t*-test unless otherwise noted. *P*-values are presented in figures and tables. To compare the difference of sIgA level between SFB-positive and SFB-negative groups, non-linear fit curves were introduced into the data using GraphPad Prism 6.

## Author Contributions

BC, HC, YY, XS, and JL performed the experiments. LC, KP, WG, HZ, YE, and LJ collected samples. FX, JQ, JS, and LL conducted data analysis. HY, MJ, XW, and CX contributed to the design of experiments and composition of the manuscript.

## Conflict of Interest Statement

The authors declare that the research was conducted in the absence of any commercial or financial relationships that could be construed as a potential conflict of interest.

## Footnotes

1 http://www.uniprot.org/

2 https://blast.ncbi.nlm.nih.gov/Blast.cgi
