# Supplementary material for: Presence of Segmented Filamentous Bacteria in Human Children and Its Potential Role in the Modulation of Human Gut Immunity
Source: Front Microbiol. 2018 Jun 29;9:1403. doi: 10.3389/fmicb.2018.01403 (PMC6034559; doi:10.3389/fmicb.2018.01403)
Supplement: Supplementary file 1 [file Data_Sheet_1.docx]

**Supplementary materials**

**Presence of segmented filamentous bacteria in human children are associationed with gut immunity**

Bo Chen^a,c*^, Huahai Chen^b*^, Yeshi Yin^b*^, Xiaoli Shu^c*^, Jia Li^a^,Junjie Qin^d^, Lijun Chen^a^,Kerong Peng^c^, Fei Xu^b^, Weizhong Gu^c^, Hong Zhao^c^, Liqin Jiang^c^, Lanjuan Li^a^, Jian Song^e^, Yoram Elitsur^f^, Hongwei D. Yu^f,g^, Mizu Jiang^c^, Xin Wang^b^, Charlie Xiang^a^

**Bioinformatics analysis and primer design**

Conserved genes among all SFB genomes were identified by sequence comparison of SFB-rat-Yit genes against all other 10 SFB genomes (complete and unfinished WGS). Any SFB-rat-Yit gene that has a significant hit (e-10) to other SFB genomes was considered as a conserved gene. A total of 1297 conserved SFB genes were identified. Of those conserved genes, 553 of them have a perfect match to at least one other SFB genome. The sequence of these conserved genes were then compared to all available genomes of *Clostridium* spp. and genes that had a significant hit to *Clostridium* genomes (e-10) were removed and finally, a total of 441 conserved, yet unique SFB genes were identified. These 441 genes were ranked based on their conservation (number of hits to other SFB genes and percent identity). Primers were designed for the top 19 conserved SFB genes using Primer3 and used to investigate the presence of SFB-like genome in human gut microflora.

**Primers, 778F/1008R were specific for SFB**

SFB specific primers, 779F /1008R, originally published by Snel’s group^1^, were used to identify SFB in mice, rats and chickens. Here, PCR products generated by primers, 779F /1008R from five luminal fluids and fecal samples were cloned into a pMD18-T vector (Takara Biotechnology Co., Ltd, Dalian, China) according to the manufacturer’s instructions to produce a shotgun library. Ten clones from each library were randomly selected and sent for sequencing. Sequences were analyzed for homology using the NCBI database. All sequences were identical and belonged to SFB. Furthermore, Josson published a 1,222 bp human SFB 16S rRNA sequence obtained from a human ileostomy sample using multiple PCR^2^. We aligned our PCR products with other SFB sequences including that of Josson and others. The sequences generated by primers 779F /1008R from all samples was identical and specific for SFB.

**Distribution of SFB in American children**

From 2014 to 2015, fifty four fecal samples were collected from the Section of Gastroenterology in the Department of Pediatrics, Joan C. Edwards School of Medicine, Marshall University. Among the samples, 41 were collected from the volunteers aged under 3 years old, 4 between 3 to 6 years old and 5 between 9 to 15 years old.DNA was extracted from stool samples using the QIAamp DNA Stool Mini Kit (Qiagen). The DNA concentrations were measured using NanoDrop1000. Approximately 100 ng DNA was used as template with primers 779F/1008R for PCR reaction with the fecal DNA of C57BL/6 mice used as the positive control. Gel-confirmed PCR products (~200 bp) were cloned into pCR®4 as part of the TOPO TA Cloning Kit (Invitrogen) for DNA sequencing. Plasmid DNA was extracted from the TOPO cloned products using a MiniPrep Plasmid Purification Kit (Qiagen). TOPO cloned products with a DNA concentration of approximately 100 ng/μL or greater were sent for DNA sequencing by the West Virginia University Genomics Core Facility using the M13F and M13R primers. The percentages of SFB from a small patient sample population from West Virginia were 68.3%, 50% and 33.33% respectively.

**Identification of SFB from metagenomics data**

With about 4 Gb of Illumina sequence data generated per sample, only 0.2% to 1.5% of known SFB genomes region were covered. We then aligned the total of Illumina sequence data of all samples onto known SFB genomes, however, the genome coverage was too low to prove the SFB existence.

**Table S1. Detailed Sample Description for analysis of the relationship between SFB and human gut immunity**

| **SAMPLE ID** | **Sex** | **Ages** | **(PCR)*** | **(SEM)^a^** | **(FISH)^b^** | **CHIPS**  n=3vs3 | **SIgA**  n=23vs  24 | **mRNA**  n=22 | **QPCR**  n=24 | **Meta**  n=7vs4 | **non-16S^d^**  n=30 |
| --- | --- | --- | --- | --- | --- | --- | --- | --- | --- | --- | --- |
|  |  | (month) | (SFB ratio=36/144) | (SFB ratio =23/54) | (SFB ratio =36/96) |  |  |  |  |  |  |
| 1 | M | 7 | – |  | – |  | M |  |  |  |  |
| 2 | F | 7 | – |  | – |  | M |  |  |  |  |
| 3 | M | 8 | + |  | + |  | M | Q | q |  | 1,2 |
| 4 | M | 12 | + | + | + |  |  |  | q |  | 1,2 |
| 5 | F | 13 | + | + | + |  | M | Q | q |  | 1,2 |
| 6 | M | 14 | – | – | – |  | M | Q |  |  |  |
| 7 | F | 15 | – |  | – |  | M | Q |  |  |  |
| 8 | M | 15 | – | – | – |  | M | Q |  |  |  |
| 9 | F | 16 | – |  |  |  |  | Q |  |  |  |
| 10 | F | 17 | – |  |  |  |  |  |  |  |  |
| 11 | M | 20 | – |  |  |  |  |  |  |  |  |
| 12 | F | 20 | – |  |  |  |  |  |  |  |  |
| 13 | M | 21 | + | + | + |  | M | Q | q | m | 1,2 |
| 14 | M | 21 | – | – | – |  |  | Q |  |  |  |
| 15 | M | 21 | + | + | + |  |  | Q | q |  | 1,2 |
| 16 | M | 22 | + | + | + |  | M | Q | q | m | 1,2 |
| 17 | F | 22 | – |  |  |  |  |  |  |  |  |
| 18 | F | 22 | – |  |  |  |  |  |  |  |  |
| 19 | F | 23 | – | – | – |  | M | Q |  |  |  |
| 20 | M | 24 | + | + | + |  | M | Q | q |  | 1,2 |
| 21 | M | 25 | + | + | + |  |  | Q | q |  | 1,2 |
| 22 | M | 25 | + | + | + |  | M | Q | q |  | 1,2 |
| 23 | F | 26 | – |  |  |  |  |  |  |  |  |
| 24 | M | 26 | – |  |  |  |  |  |  |  |  |
| 25 | F | 27 | + | + | + | C | M | Q | q | m | 1,2 |
| 26 | M | 27 | + | + | + | C |  | Q | q | m | 1,2 |
| 27 | F | 27 | – | – | – | C | M | Q |  | m |  |
| 28 | M | 27 | – | – | – | C | M | Q |  | m |  |
| 29 | M | 28 | + |  | + | C | M |  | q | m | 1,2 |
| 30 | M | 28 | – | – | – |  |  |  |  | m |  |
| 31 | M | 28 | + |  | + |  |  | Q | q | m | 1,2 |
| 32 | F | 28 | – | – | – |  | M |  |  | m |  |
| 33 | M | 29 | – |  |  |  |  |  |  |  |  |
| 34 | M | 29 | + |  | + |  | M |  | q | m | 1,2 |
| 35 | M | 29 | – |  |  |  |  |  |  |  |  |
| 36 | F | 29 | + |  | + |  | M |  |  |  | 1,2 |
| 37 | M | 30 | – |  |  |  |  |  |  |  |  |
| 38 | F | 30 | – | – | – |  |  |  |  |  |  |
| 39 | F | 30 | – | – | – | C | M |  |  |  |  |
| 40 | M | 31 | – |  |  |  |  |  |  |  |  |
| 41 | M | 31 | – |  |  |  |  |  |  |  |  |
| 42 | M | 31 | – |  |  |  |  |  |  |  |  |
| 43 | F | 32 | + | + | + |  | M |  | q |  | 1,2 |
| 44 | F | 32 | – |  |  |  |  |  |  |  |  |
| 45 | M | 32 | – |  |  |  |  |  |  |  |  |
| 46 | M | 32 | + | + | + |  | M |  | q |  | 1,2 |
| 47 | F | 33 | – |  |  |  |  | Q |  |  |  |
| 48 | M | 34 | + |  | + |  |  |  |  |  | 1,2 |
| 49 | F | 34 | – |  |  |  |  | Q |  |  |  |
| 50 | M | 34 | + | + | + |  |  |  | q |  | 1,2 |
| 51 | F | 35 | – |  |  |  |  |  |  |  |  |
| 52 | M | 36 | – |  |  |  |  |  |  |  |  |
| 53 | F | 36 | + | + | + |  |  |  | q |  | 1,2 |
| 54 | M | 36 | + | + | + |  | M |  | q |  | 1,2 |
| 55 | M | 36 | + |  | + |  | M |  |  |  | 2 |
| 56 | F | 36 | – |  |  |  |  | Q |  |  |  |
| 57 | M | 36 | – |  |  |  |  |  |  |  |  |
| 58 | F | 36 | – |  |  |  |  |  |  |  |  |
| 59 | F | 36 | + | + | + |  | M |  | q |  | 1,2 |
| 60 | M | 37 | – |  |  |  |  |  |  |  |  |
| 61 | M | 40 | – |  |  |  |  |  |  |  |  |
| 62 | M | 41 | – |  |  |  |  |  |  |  |  |
| 63 | F | 42 | – |  | – |  |  |  |  |  |  |
| 64 | F | 42 | + | + | + |  | M |  | q |  | 1,2 |
| 65 | F | 42 | – |  | - |  | M |  |  |  |  |
| 66 | F | 42 | – |  |  |  |  |  |  |  |  |
| 67 | F | 42 | – |  |  |  |  |  |  |  |  |
| 68 | F | 44 | + |  | + |  | M |  | q |  | 1,2 |
| 69 | F | 44 | – |  | – |  | M |  |  |  |  |
| 70 | M | 44 | – |  |  |  |  |  |  |  |  |
| 71 | M | 47 | – |  | – |  | M |  |  |  |  |
| 72 | M | 47 | – |  |  |  |  |  |  |  |  |
| 73 | M | 48 | + |  | + |  | M |  | q |  | 1,2 |
| 74 | F | 48 | + | + | + |  | M |  | q |  | 1,2 |
| 75 | M | 48 | – |  | – |  | M |  |  |  |  |
| 76 | F | 48 | – | – | – |  |  |  |  |  |  |
| 77 | F | 49 | + |  | + |  | M |  |  |  | 1,2 |
| 78 | M | 50 | – |  |  |  |  |  |  |  |  |
| 79 | F | 51 | – |  | – |  | M |  |  |  |  |
| 80 | F | 54 | + |  | + |  | M |  |  |  | 1,2 |
| 81 | M | 54 | – |  | – |  |  |  |  |  |  |
| 82 | M | 57 | – | – | – |  | M |  |  |  |  |
| 83 | M | 57 | + | + | + |  | M |  |  |  | 1,2 |
| 84 | M | 57 | – | – | – |  |  |  |  |  |  |
| 85 | F | 60 | + | + | + |  |  |  |  |  | 1,2 |
| 86 | F | 60 | – | – | – |  | M |  |  |  |  |
| 87 | F | 60 | – | – | - |  | M |  |  |  |  |
| 88 | F | 64 | – |  | – |  |  |  |  |  |  |
| 89 | M | 64 | – | – | – |  | M |  |  |  |  |
| 90 | M | 66 | – |  | – |  |  |  |  |  |  |
| 91 | M | 66 | – |  |  |  |  |  |  |  |  |
| 92 | M | 66 | – |  | – |  | M |  |  |  |  |
| 93 | M | 67 | – |  |  |  |  |  |  |  |  |
| 94 | M | 68 | – |  | – |  | M |  |  |  |  |
| 95 | M | 69 | + |  | + |  | M |  |  |  |  |
| 96 | M | 72 | – |  |  |  |  |  |  |  |  |
| 97 | M | 72 | – | – | – |  | M |  |  |  |  |
| 98 | M | 72 | – |  |  |  |  |  |  |  |  |
| 99 | F | 72 | – |  |  |  |  |  |  |  |  |
| 100 | F | 72 | – |  |  |  |  |  |  |  |  |
| 101 | M | 73 | – |  | – |  | M |  |  |  |  |
| 102 | F | 74 | – | – | – |  | M |  |  |  |  |
| 103 | M | 75 | – |  | – |  |  |  |  |  |  |
| 104 | F | 75 | – |  | – |  |  |  |  |  |  |
| 105 | M | 76 | – |  | – |  |  |  |  |  |  |
| 106 | F | 77 | – |  |  |  |  |  |  |  |  |
| 107 | M | 78 | – |  | – |  |  |  |  |  |  |
| 108 | M | 78 | – |  |  |  |  |  |  |  |  |
| 109 | F | 79 | + | + | + |  |  |  |  |  |  |
| 110 | F | 79 | – |  | – |  |  |  |  |  |  |
| 111 | F | 79 | – | – | – |  |  |  |  |  |  |
| 112 | F | 79 | – |  | – |  |  |  |  |  |  |
| 113 | M | 80 | – |  | – |  |  |  |  |  |  |
| 114 | F | 81 | + | + | + |  |  |  |  |  |  |
| 115 | M | 82 | – | – | – |  |  |  |  |  |  |
| 116 | F | 83 | – | – | – |  |  |  |  |  |  |
| 117 | M | 84 | – |  | – |  |  |  |  |  |  |
| 118 | M | 84 | – | – | – |  |  |  |  |  |  |
| 119 | F | 84 | – |  | – |  |  |  |  |  |  |
| 120 | M | 91 | – |  | – |  |  |  |  |  |  |
| 121 | M | 97 | – |  |  |  |  |  |  |  |  |
| 122 | F | 98 | – | – | – |  |  |  |  |  |  |
| 123 | M | 100 | – | – | – |  |  |  |  |  |  |
| 124 | F | 108 | – | – | – |  |  |  |  |  |  |
| 125 | M | 108 | + | + | + |  |  |  |  |  |  |
| 126 | M | 108 | – |  | – |  |  |  |  |  |  |
| 127 | F | 108 | – | – | – |  |  |  |  |  |  |
| 128 | F | 109 | – |  |  |  |  |  |  |  |  |
| 129 | M | 109 | – | – | – |  |  |  |  |  |  |
| 130 | F | 114 | – |  | – |  |  |  |  |  |  |
| 131 | M | 121 | + | + | + |  |  |  |  |  |  |
| 132 | F | 126 | – | – | – |  |  |  |  |  |  |
| 133 | F | 127 | – | – | – |  |  |  |  |  |  |
| 134 | M | 128 | – |  |  |  |  |  |  |  |  |
| 135 | F | 137 | – |  |  |  |  |  |  |  |  |
| 136 | M | 139 | – |  | – |  |  |  |  |  |  |
| 137 | F | 142 | – |  |  |  |  |  |  |  |  |
| 138 | M | 144 | – |  |  |  |  |  |  |  |  |
| 139 | M | 144 | – | – | – |  |  |  |  |  |  |
| 140 | F | 152 | – |  |  |  |  |  |  |  |  |
| 141 | M | 156 | – |  | – |  |  |  |  |  |  |
| 142 | M | 174 | – |  |  |  |  |  |  |  |  |
| 143 | F | 174 | – |  |  |  |  |  |  |  |  |
| 144 | M | 178 | – |  | – |  |  |  |  |  |  |

^a^: examined by SEM;

^b^: determined by FISH with SFB specific probe;

^c^: diagnosed by colonoscopy and pathological examination;

^d^: detected by non-16s SFB primers, 1, primer SFB330-13. 2, primer SFB8576

Ms,m,Q,q,C means samples mesured.

+: SFB positive;

-: SFB negative.

**Table S2. Primer sequences and probe used in this study**

| **Name** | **Sequences** |
| --- | --- |
| Total bacteria Bac1114F/1275R | 5'-CGGCAACGAGCGCAACCC-3'/5'CCATTGTAGCACGTGTGTAGCC-3' |
| SFB specific primers 779/1008  SFB specific primers 779/1380 | 5'-TGTGGGTTGTGAATAACAAT-3'/5'-GCGGGCTTCCCTCATTACAAGG-3'  5'-TGTGGGTTGTGAATAACAAT-3'/5'-GGTTAGCCCACAGGCTTCGG-3' |
| SFB specific probe  SFB330-13  SFB123-14  SFB8576  SFB10797  SFB10788  SFB130710  SFB81511  R94716  R49017  SFB123-21  SFB330-22  SFB7605  SFB8612  SFB2613  SFBM2114  SFBM3515  R36218  R107419  R27320 | GCGGGCTTCCCTCATTACAAGG  5'-GTAGTACAGATTATAGATATTT-3'/5'-TATCAACCTTTATAATATTACT-3'  5'-ATTTTAATGAATGGGATGATAGA-3'/5'-CAAGAAAACTTCCATCACACCATCT-3'  5'-GAATTTTTTGCGAACAATATTTT-3'/5'-TGAAGTCGCAACTATATTTTCAT-3'  5'-AATTAAGGATGGTATATTCCATGA-3'/5'-TATTTTTTAAAGTTTATTTCT-3'  5'-ATCTTCCGTAGATTCTAAAGACAGA-3'/5'-CGCATTATCATTTGTTGTCCATA-3'  5'-GGATCTTCAGTCGTACTAACTGGA-3'/5'-ATTATTTTCAAAATTCAATAA-3'  5'- TGAATGAAGATGAAATAAATAAGATT-3'/5'- AGGACCCATATATTCAATTTCTTCACG-3'  5'-ATGATTAGAGGAATGTATACAGCTAT-3'/5'- TTAAACTTTCCCAATATCATT-3'  5'-ATGAATGGTGAAAAATTAATATTA-3'/5'- TTACTTTAAATTCAAATTGAAAAATCTATCCAT-3'  5'-TGTTTGAATATAAAAGAGCCT-3'/5'-TGTAATAAGAACTGAATTAT-3'  5'-TAGCTGGAGATAATATTTATTTA-3'/5'-ACTGAAATACCCATGACCACCATCA-3'  5'-CATTAGTGACTATGTTTATATT-3'/5'-AATGCTCTGACTGACCAGAAGTCCT-3'  5'-ATAGTAAAAATTGAGGGTTA-3'/5'-TTAACTCCACTATTTACTAAATTA-3'  5'-TGTTTAATGGTATTCTCTAGT-3'/5'-TCAATAACTATAAATATAAAAA-3'  5'-AGAGGGCGGACTTATAGTTGA-3'/5'-AGTATCCTTTTTCTGCCA-3'  5'-TGCATCTTTAGTAGCTGCATTTTA-3'/5'-AGAATTAAGTACTGCTGGAGT-3'  5'-ATGAGAAAGAAGAAATTAATTAGTATTTT-3'/5'-CTATTCGATTGAAATCTTTTCTAAGT-3'  5'-ATGTTTAGTAAAAAATTTATGAAGAAA-3'/5'-TTACTCTACTCTTAATCCAAGACTA-3'  5'-TTGGCTAGCAATAAAAAATCTAA-3'/5'-TTACGGATTTACACCAATCCT-3' |
| TNFα F/R | 5'-CAAGACCACCACTTCGAAACCT-3'/5'-TTAGTGGTTGCCAGCACTTCA-3' |
|  |  |
| CD3E F/R | 5'-CCCCATCCCAAAGTATTCCA-3'/5'-CCCAGTCCATCCCCAGAGA-3' |
|  |  |
| IFNγF/R | 5'-CCAACGCAAAGCAATACATGA-3'/5'-TTTTCGCTTCCCTGTTTTAGCT-3' |
|  |  |
| FOXP3 F/R | 5'-ATCCGCCACAACCTGAGTCT-3'/5'-TCCACACAGCCCCCTTCTC-3' |
| CXCL10 F/R | 5'-AAACCAGAGGGGAGCAAAAT-3'/5'-TAGGGAAGTGATGGGAGAGG-3' |
| IL17 F/R | 5'-TCCTAGGGCCTGGCTTCTG-3'/5'-AGTTCGTTCTGCCCCATCAG-3' |
| IL10 F/R | 5'-GGGAGCCCCTTTGATGATTAA-3'/5'-GCCACAGCTTTCAAGAATGAAGT-3' |
| Fut2 F/R | 5'-TCCGATGGGTAGGAATTGTCA-3'/5'-GGCTAAACTAATCCCAGAAGTCTCA-3' |
| SAA F/R | 5'-GCTGATCAGGCTGCCAATG-3'/5'-GCCAGCAGGTCGGAAGTG-3' |

**Table S3: Genes statistically regulated (P<0.05) between SFB positive and negative group.** mRNAs were extracted from issue biopsy specimens (SFB positive samples n=3, negative samples n=3) and gene expression were analyzed using Agilent Whole Human Genome Oligo Microarray. Data were compared by two groups (SFB positive vs SFB negative).

| Number |  |  | Name |  |
| --- | --- | --- | --- | --- |
| 1 | A-23-P411612 | NM-207344 | SPRYD4 | SPRY domain containing 4 |
| 2 | A-23-P254896 | NM-003868 | FGF16 | Fibroblast grown factor 16 |
| 3 | A-23-P427014 | NM-199328 | CLDN8 | Claudin8 |
| 4 | A-24-P655458 | NM-198153 | TREML4 | Triggering receptor expressed on myeloid cells-like 4 |
| 5 | A-33-P3267769 | NM-001001914 | OR2G3 | Olfactory receptor, family 2, subfamily G, member 3 |
| 6 | A-19-P00800080 |  |  |  |

| Microarray sample ID | 1vs.6 | 1vs.7 | 1vs.8 | 2vs.6 | 2vs.7 | 2vs.8 | 3vs.6 | 3vs.7 | 3vs.8 |
| --- | --- | --- | --- | --- | --- | --- | --- | --- | --- |
| BCR | **+** | **+** | **+** | **+** |  |  | **+** | **+** | **+** |
| ID-hsa04978 | **+** | **+** | **+** |  |  |  |  |  |  |
| IL4 | **+** | **+** |  | **+** | **+** |  | **+** |  |  |
| IL7 | **+** |  |  |  |  |  |  |  |  |
| KitReceptor | **+** | **+** | **+** |  |  |  |  |  |  |
| TCR | **+** | **+** | **+** | **+** | **+** | **+** |  | **+** |  |
| MT-HeavyMetal | **+** | **+** | **+** | **+** | **+** | **+** | **+** | **+** | **+** |
| a6b4Integrin |  | **+** |  |  |  |  |  |  |  |
| EGFR1 |  | **+** |  |  |  |  |  |  |  |

**Table S4: The statistically significant up-regulated (P<0.05) pathways between SFB positive person and SFB negative person.** mRNAs were extracted from issue biopsy specimens (SFB positive samples n=3, negative samples n=3) and gene expression were analyzed using Agilent Whole Human Genome Oligo Microarray. Data were compared by person to person.

SFB positive number: 1, 2, 3; SFB negative number: 6, 7, 8.

**Table S5: An example of T and B cell signaling pathway genes activated in human terminal ileal biopsies in response to SFB colonization.**  These pathways were identified in the majority of person to person microarray comparison.

| Gene Symbol | Description | P value |
| --- | --- | --- |
| ABL1 | proto-oncogene 1, non-receptor tyrosine kinase] [Gene Type: protein-coding] | 0.000028229 |
| ACP1 | acid phosphatase 1, soluble] [Gene Type: protein-coding] | 0.000036336 |
| AKT1 | AKT serine/threonine kinase 1] [Gene Type: protein-coding] | 0.00000367 |
| ARHGDIB | Rho GDP dissociation inhibitor beta] [Gene Type: protein-coding] | 0.000037633 |
| ARHGEF6 | Rac/Cdc42 guanine nucleotide exchange factor 6] [Gene Type: protein-coding] | 0.000000919 |
| ARHGEF7 | Rho guanine nucleotide exchange factor 7] [Gene Type: protein-coding] | 0.000039373 |
| Bcl-6 | B-cell CLL/lymphoma 6 | 0.00005734 |
| BLNK | B-cell linker ( BLNK ) | 0.000035473 |
| CARD11 | caspase recruitment domain family member 11] [Gene Type: protein-coding] | 0.000002211 |
| CBL | Cbl proto-oncogene] [Gene Type: protein-coding] | 0.000031229 |
| CBLB | Cbl proto-oncogene B] [Gene Type: protein-coding] | 0.000043877 |
| CD2 | CD2 molecule] [Gene Type: protein-coding] | 0.00000672 |
| CD2AP | CD2 associated protein] [Gene Type: protein-coding] | 0.000040682 |
| CD3D | CD3d molecule] [Gene Type: protein-coding] | 0.000008461 |
| CD3E | CD3e molecule] [Gene Type: protein-coding] | 0.000042423 |
| CD3G | CD3g molecule] [Gene Type: protein-coding] | 0.000005217 |
| CD4 | CD4 molecule] [Gene Type: protein-coding] | 0.00004367 |
| CD5 | CD5 molecule] [Gene Type: protein-coding] | 0.000006957 |
| CD8A | CD8a molecule] [Gene Type: protein-coding] | 0.000018701 |
| CD19 | CD19 molecule] [Gene Type: protein-coding] | 0.000023585 |
| CD79a | CD79a molecule] [Gene Type: protein-coding] | 0.000021234 |
| CD81 | CD81 molecule] [Gene Type: protein-coding] | 0.000017234 |
| CD247 | CD247 molecule] [Gene Type: protein-coding] | 0.000003259 |
| CDC42 | cell division cycle 42] [Gene Type: protein-coding] | 0.000010966 |
| CEBPB | CCAAT/enhancer binding protein beta] [Gene Type: protein-coding] | 0.000000518 |
| CISH | cytokine inducible SH2 containing protein] [Gene Type: protein-coding] | 0.000012258 |
| CREB1 | cAMP responsive element binding protein 1] [Gene Type: protein-coding] | 0.00000181 |
| CREBBP | CREB binding protein] [Gene Type: protein-coding] | 0.000013999 |
| CRK | CRK proto-oncogene, adaptor protein] [Gene Type: protein-coding] | 0.000021697 |
| CRKL | CRK like proto-oncogene, adaptor protein] [Gene Type: protein-coding] | 0.000010804 |
| CTNNB1 | catenin beta 1] [Gene Type: protein-coding] | 0.000023443 |
| DBNL | drebrin like] [Gene Type: protein-coding] | 0.000007505 |
| DEF6 | DEF6, guanine nucleotide exchange factor] [Gene Type: protein-coding] | 0.000015258 |
| DEFB4 | defensin beta 104A] [Gene Type: protein-coding]; 1673 [Gene Symbol: DEFB4A] [Locus Tag: ] [Chromosome: 8] [Map Location: 8p23.1] [Description: defensin beta 4A] [Gene Type: protein-coding] | 0.000008801 |
| DLG1 | discs large MAGUK scaffold protein 1] [Gene Type: protein-coding] | 0.000016999 |
| DNM2 | dynamin 2] [Gene Type: protein-coding] | 0.000011852 |
| DOCK2 | dedicator of cytokinesis 2] [Gene Type: protein-coding] | 0.000024494 |
| DUSP3 | dual specificity phosphatase 3] [Gene Type: protein-coding] | 0.000031699 |
| ELK4 | ELK4, ETS transcription factor] [Gene Type: protein-coding] | 0.000021295 |
| ENAH | enabled homolog (Drosophila)] [Gene Type: protein-coding] | 0.000033439 |
| EVL | Enah/Vasp-like] [Gene Type: protein-coding] | 0.000024333 |
| FCRL3 | Fc receptor like 3] [Gene Type: protein-coding] | 0.000031537 |
| FOS | Fos proto-oncogene, AP-1 transcription factor subunit] [Gene Type: protein-coding] | 0.000044229 |
| FYB | FYN binding protein] [Gene Type: protein-coding] | 0.000025142 |
| FYN | FYN proto-oncogene, Src family tyrosine kinase] [Gene Type: protein-coding] | 0.000037736 |
| GAB2 | GRB2 associated binding protein 2] [Gene Type: protein-coding] | 0.000026834 |
| GIT2 | GIT ArfGAP 2] [Gene Type: protein-coding] | 0.000039028 |
| GRAP | GRB2-related adaptor protein] [Gene Type: protein-coding] | 0.000002319 |
| GRAP2 | GRB2-related adaptor protein 2] [Gene Type: protein-coding] | 0.000036282 |
| GRB2 | growth factor receptor bound protein 2] [Gene Type: protein-coding] | 0.000003616 |
| HDAC7 | histone deacetylase 7] [Gene Type: protein-coding] | 0.000032589 |
| HOMER3 | homer scaffolding protein 3] [Gene Type: protein-coding] | 0.000040786 |
| ITK | IL2 inducible T-cell kinase] [Gene Type: protein-coding] | 0.000034374 |
| ITPR1 | inositol 1,4,5-trisphosphate receptor type 1] [Gene Type: protein-coding] | 0.000042082 |
| JAK3 | Janus kinase 3] [Gene Type: protein-coding] | 0.000009312 |
| JUN | Jun proto-oncogene, AP-1 transcription factor subunit] [Gene Type: protein-coding] | 0.000043823 |
| KHDRBS1 | KH RNA binding domain containing, signal transduction associated 1] [Gene Type: protein-coding] | 0.000006117 |
| LAT | linker for activation of T-cells] [Gene Type: protein-coding] | 0.000040574 |
| LAX1 | lymphocyte transmembrane adaptor 1] [Gene Type: protein-coding] | 0.000007858 |
| LCP2 | lymphocyte cytosolic protein 2] [Gene Type: protein-coding] | 0.000037334 |
| LIME1 | Lck interacting transmembrane adaptor 1] [Gene Type: protein-coding] | 0.000004164 |
| LYN | LYN proto-oncogene, Src family tyrosine kinase] [Gene Type: protein-coding] | 0.000012361 |
| MAP2K1 | mitogen-activated protein kinase kinase 1] [Gene Type: protein-coding] | 0.000001414 |
| MAP4K1 | mitogen-activated protein kinase kinase kinase kinase 1] [Gene Type: protein-coding] | 0.000013653 |
| MAPK1 | mitogen-activated protein kinase 1] [Gene Type: protein-coding] | 0.000003209 |
| MARS | methionyl-tRNA synthetase] [Gene Type: protein-coding] | 0.00000672 |
| MATK | megakaryocyte-associated tyrosine kinase] [Gene Type: protein-coding] | 0.000040682 |
| MUC1 | mucin 1, cell surface associated] [Gene Type: protein-coding] | 0.000008461 |
| NCK1 | NCK adaptor protein 1] [Gene Type: protein-coding] | 0.000042423 |
| NCL | nucleolin] [Gene Type: protein-coding] | 0.000005217 |
| NEDD9 | neural precursor cell expressed, developmentally down-regulated 9] [Gene Type: protein-coding] | 0.00004367 |
| NFAM1 | NFAT activating protein with ITAM motif 1] [Gene Type: protein-coding] | 0.000006957 |
| NFATC2 | nuclear factor of activated T-cells 2] [Gene Type: protein-coding] | 0.000018701 |
| PAG1 | phosphoprotein membrane anchor with glycosphingolipid microdomains 1] [Gene Type: protein-coding] | 0.000003259 |
| PAK1 | p21 (RAC1) activated kinase 1] [Gene Type: protein-coding] | 0.000010966 |
| PKN1 | protein kinase N1] [Gene Type: protein-coding] | 0.000000518 |
| PLCG1 | phospholipase C gamma 1] [Gene Type: protein-coding] | 0.000012258 |
| PPP3CB | protein phosphatase 3 catalytic subunit beta] [Gene Type: protein-coding] | 0.00000181 |
| PRKCQ | protein kinase C theta] [Gene Type: protein-coding] | 0.000013999 |
| PRKD2 | protein kinase D2] [Gene Type: protein-coding] | 0.000021697 |
| PSAP | prosaposin] [Gene Type: protein-coding] | 0.000010804 |
| PSTPIP1 | proline-serine-threonine phosphatase interacting protein 1] [Gene Type: protein-coding] | 0.000023443 |
| PTK2 | protein tyrosine kinase 2] [Gene Type: protein-coding] | 0.000007505 |
| PTK2B | protein tyrosine kinase 2 beta] [Gene Type: protein-coding] | 0.000015258 |
| PTPN3 | protein tyrosine phosphatase, non-receptor type 3] [Gene Type: protein-coding] | 0.000008801 |
| PTPN6 | protein tyrosine phosphatase, non-receptor type 6] [Gene Type: protein-coding] | 0.000016999 |
| PTPN12 | protein tyrosine phosphatase, non-receptor type 12] [Gene Type: protein-coding] | 0.000029198 |
| PTPN22 | protein tyrosine phosphatase, non-receptor type 22] [Gene Type: protein-coding] | 0.000018295 |
| PTPRC | protein tyrosine phosphatase, receptor type C] [Gene Type: protein-coding] | 0.000026448 |
| PTPRJ | protein tyrosine phosphatase, receptor type J] [Gene Type: protein-coding] | 0.000015096 |
| PXN | paxillin] [Gene Type: protein-coding] | 0.000027784 |
| RAP1A | RAP1A, member of RAS oncogene family] [Gene Type: protein-coding] | 0.000011852 |
| RASA1 | RAS p21 protein activator 1] [Gene Type: protein-coding] | 0.000024494 |
| RASGRF1 | Ras protein specific guanine nucleotide releasing factor 1] [Gene Type: protein-coding] | 0.000031699 |
| RASGRP2 | RAS guanyl releasing protein 2] [Gene Type: protein-coding] | 0.000021295 |
| RIPK2 | receptor interacting serine/threonine kinase 2] [Gene Type: protein-coding] | 0.000016999 |
| SH2D2A | SH2 domain containing 2A] [Gene Type: protein-coding] | 0.000029198 |
| SH2D3C | SH2 domain containing 3C] [Gene Type: protein-coding] | 0.000018295 |
| SH3BP2 | SH3 domain binding protein 2] [Gene Type: protein-coding] | 0.000026448 |
| SHB | SH2 domain containing adaptor protein B] [Gene Type: protein-coding] | 0.000015096 |
| SHC1 | SHC adaptor protein 1] [Gene Type: protein-coding] | 0.000027784 |
| SIT1 | signaling threshold regulating transmembrane adaptor 1] [Gene Type: protein-coding] | 0.000011852 |
| SKAP1 | src kinase associated phosphoprotein 1] [Gene Type: protein-coding] | 0.000024494 |
| SKAP2 | src kinase associated phosphoprotein 2] [Gene Type: protein-coding] | 0.000031699 |
| SLA | Src-like-adaptor] [Gene Type: protein-coding] | 0.000021295 |
| SLA2 | Src like adaptor 2] [Gene Type: protein-coding] | 0.000033439 |
| SOS1 | SOS Ras/Rac guanine nucleotide exchange factor 1] [Gene Type: protein-coding] | 0.000022587 |
| SOS2 | SOS Ras/Rho guanine nucleotide exchange factor 2] [Gene Type: protein-coding] | 0.000030245 |
| SRC | SRC proto-oncogene, non-receptor tyrosine kinase] [Gene Type: protein-coding] | 0.000024333 |
| STAT5A | signal transducer and activator of transcription 5A] [Gene Type: protein-coding] | 0.000031537 |
| STAT5B | signal transducer and activator of transcription 5B] [Gene Type: protein-coding] | 0.000044229 |
| STK39 | serine/threonine kinase 39] [Gene Type: protein-coding] | 0.000028337 |
| SYK | spleen associated tyrosine kinase] [Gene Type: protein-coding] | 0.000036045 |
| SYP | synaptophysin] [Gene Type: protein-coding] | 0.000025142 |
| TRAT1 | T cell receptor associated transmembrane adaptor 1] [Gene Type: protein-coding] | 0.000037736 |
| TRB@ | T cell receptor beta locus] [Gene Type: protein-coding] | 0.000026834 |
| TUBA4A | tubulin alpha 4a] [Gene Type: protein-coding] | 0.000039028 |
| TUBB | tubulin beta class I] [Gene Type: protein-coding] | 0.000002319 |
| TXK | TXK tyrosine kinase] [Gene Type: protein-coding] | 0.000036282 |
| UNC119 | unc-119 lipid binding chaperone] [Gene Type: protein-coding] | 0.000003616 |
| VASP | vasodilator-stimulated phosphoprotein] [Gene Type: protein-coding] | 0.000032589 |
| VAV1 | vav guanine nucleotide exchange factor 1] [Gene Type: protein-coding] | 0.000040786 |
| VAV3 | vav guanine nucleotide exchange factor 3] [Gene Type: protein-coding] | 0.000034374 |
| WAS | Wiskott-Aldrich syndrome] [Gene Type: protein-coding] | 0.000042082 |
| WASF2 | WAS protein family member 2] [Gene Type: protein-coding] | 0.000009312 |
| WIPF1 | WAS/WASL interacting protein family member 1] [Gene Type: protein-coding] | 0.00007077 |
| YWHAQ | tyrosine 3-monooxygenase/tryptophan 5-monooxygenase activation protein theta] [Gene Type: protein-coding] | 0.00006661 |
| ZAP70 | zeta chain of T cell receptor associated protein kinase 70] [Gene Type: protein-coding] | 0.00000873 |

**Figure S1**


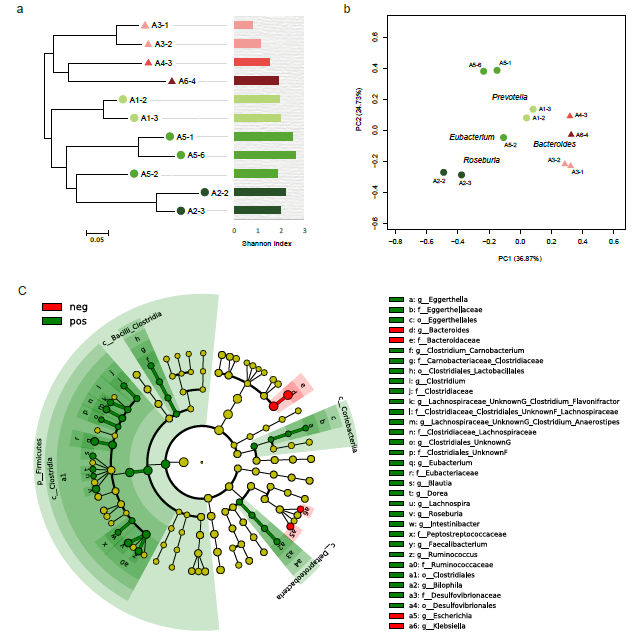


**Figure S1. Metagenomic analysis of the gut microbiota.** About 500 μg of gut contents from each patient was used for the extraction of genomic DNA (a total of 11 samples). About 2 μg amount of genomic DNA from each patient was used for de novo sequencing. **a**, clustering tree based on Jensen-Shannon Divergence (JSD) distance using genus profile separates SFB positive samples (filled circles) and SFB negative samples (filled trangles) into two obvious branches. Shannon index of the former group shows higher alpha diversity, indicating a more abundant microbiota in this group. Samples with the same color come from the same person. **b**, principal coordinate analysis (PCA) of the genus profile. The top four genera as the main contributors were determined and plotted by their loadings in these two components. **c**, cladogram generated from LEfSe analysis, showing the most differentially abundant taxa enriched in microbiota from SFB positive samples (green) or SFB negative samples (red). Enriched taxa are indicated with a positive or negative LDA score, respectively (taxa with LDA score >2 and significance of a < 0.05 determined by Wilcoxon signed-rank test).

**Reference**

1. Snel J, Heinen PP, Blok HJ, Carman RJ, Duncan AJ, Allen PC, et al. Comparison of 16S rRNA sequences of segmented filamentous bacteria isolated from mice, rats, and chickens and proposal of "Candidatus Arthromitus". Int J Syst Bacteriol. 1995; **45**(4): 780-2.

2. Jonsson H. Segmented filamentous bacteria in human ileostomy samples after high-fiber intake. FEMS Microbiol Lett. 2013; **342**(1): 24-9.
